# Supplementary material for: Behavioral and Psychological Symptoms of Dementia (BPSD): Clinical Characterization and Genetic Correlates in an Italian Alzheimer’s Disease Cohort
Source: J Pers Med. 2020 Aug 14;10(3):90. doi: 10.3390/jpm10030090 (PMC7563608; doi:10.3390/jpm10030090)
Supplement: Supplementary file 1 [file jpm-10-00090-s001.pdf]

Table S1. Univariate Analysis of Variance in our BPSD cohort: Tests of Between-Subjects Effects for Gender variable and BPSD endophenotypes

Dependent Variable: **Psychosis**					
Source	Type III Sum of Squares	df	Mean Square	F	p-value
Corrected Model	263.0[a]	1	263.0	1.5	0.223
Intercept	97533	1	97532.5	553.3	0
Gender	263	1	263.0	1.5	0.223
Error	53760	305	176.3		
Total	159260	307			
Corrected Total	54023	306			
a. R Squared = 0.005 (Adjusted R Squared = 0.002)					
Dependent Variable: **Hyperactivity**					
Source	Type III Sum of Squares	df	Mean Square	F	p-value
Corrected Model	169.7[a]	1	169.7	2.0	0.155
Intercept	37506.1	1	37506.1	449.0	0
Gender	169.7	1	169.7	2.0	0.155
Error	25562.9	306	83.5		
Total	65847.0	308			
Corrected Total	25732.6	307			
a. R Squared = 0.007 (Adjusted R Squared = 0.003)					
Dependent Variable: **Moods**					
Source	Type III Sum of Squares	df	Mean Square	F	p-value
Corrected Model	410.0[a]	1	410.0	4.7	0.032
Intercept	16003.8	1	16003.8	181.8	0
**Gender**	**410.0**	**1**	**410.0**	**4.7**	**0.032**
Error	26757.1	304	88.0		
Total	47008.0	306			
Corrected Total	27167.2	305			
a. R Squared = 0.015 (Adjusted R Squared = 0.012)					
Dependent Variable: **Frontal**					
Source	Type III Sum of Squares	df	Mean Square	F	p-value.
Corrected Model	13.2[a]	1	13.2	1.8	0.185
Intercept	263.3	1	263.3	35.4	0
Gender	13.2	1	13.2	1.8	0.185
Error	2269.1	305	7.4		
Total	2620.0	307			
Corrected Total	2282.3	306			
a. R Squared = 0.006 (Adjusted R Squared = 0.003)					

Table S2. Univariate Analysis of Variance in our BPSD cohort: Tests of Between-Subjects Effects for Age variable and BPSD endophenotypes

Dependent Variable: **Psychosis**					
Source	Type III Sum of Squares	df	Mean Square	F	Sig.
Corrected Model	0.3a	1	0.3	0.0	0.968
Intercept	718.3	1	718.3	4.1	0.045
Age	0.3	1	0.3	0.0	0.968
Error	54022.4	305	177.1		
Total	159260.0	307			
Corrected Total	54022.7	306			
a. R Squared = 0.000 (Adjusted R Squared = -0.003)					
Dependent Variable: **Hyperactivity**					
Source	Type III Sum of Squares	df	Mean Square	F	Sig.
Corrected Model	85.3[a]	1	85.3	1.0	0.314
Intercept	693.3	1	693.3	8.3	0.004
Age	85.3	1	85.3	1.0	0.314
Error	25647.4	306	83.8		
Total	65847.0	308			
Corrected Total	25732.6	307			
a. R Squared = 0.003 (Adjusted R Squared = 0.000)					
Dependent Variable: **Moods**					
Source	Type III Sum of Squares	df	Mean Square	F	Sig.
Corrected Model	11.5[a]	1	11.5	0.1	0.720
Intercept	232.9	1	232.9	2.6	0.107
Age	11.5	1	11.5	0.1	0.720
Error	27155.7	304	89.3		
Total	47008.0	306			
Corrected Total	27167.2	305			
a. R Squared = 0.000 (Adjusted R Squared = -0.003)					
Dependent Variable: **Frontal**					
Source	Type III Sum of Squares	df	Mean Square	F	Sig.
Corrected Model	4.1[a]	1	4.1	0.5	0.461
Intercept	12.7	1	12.7	1.7	0.194
Age	4.1	1	4.1	0.5	0.461
Error	2278.2	305	7.5		
Total	2620.0	307			
Corrected Total	2282.3	306			
a. R Squared = 0.002 (Adjusted R Squared = -0.001)					

Table S3. Hosmer-Lemeshow goodness of fit test in single BSPD symptoms

Symptoms	Chi-Square	df	p-value
Agitation	40.441	35	0.243
Irritability	52.031	36	0.041
Night-time behavior disturbances	41.897	36	0.230
Aberrant motor behavior	28.209	35	0.785
Apathy	36.047	36	0.466
Delusions	35.088	35	0.464
Anxiety	42.725	36	0.205
Depression	36.287	36	0.455
Hallucination	27.670	36	0.839
Appetite and eating disturbances	38.262	35	0.324
Disinhibition	31.641	36	0.676
Euphoria	15.564	35	0.998

Nominal Regression, Stepwise Method: Forward Stepwise: Pearson chi-square analysis

Table S4. Stepwise multiple logistic regression for “Psychosis” endophenotype

Node 1.

Descriptive Statistics

	Mean	Std. Deviation	N
Psychosis	18.51	13.287	307
APOEcarE4	0.53	0.500	307
MTHFR_677T-carriers	0.31	0.462	307
MTHFR_677C-carriers	0.17	0.379	307
MTHFR_1298A-carriers	0.09	0.288	307
MTHFR_1298C-carriers	0.48	0.500	307
COMT_V158M-A-cariers	0.28	0.448	307
COMT_V158M-G-cariers	0.20	0.402	307

Variables Entered/Removed[a]

Model	Variables Entered	Variables Removed	Method
1	APOEcarE4	.	Stepwise (Criteria: Probability-of-F-to-enter <= 0.050. Probability-of-F-to-remove >= 0.100).

a. Dependent Variable: Psychosis

Model Summary

Model	R	R Square	Adjusted R Square	Std. Error of the Estimate	Change Statistics				
					R Square Change	F Change	df1	df2	Sig. F Change
1	0.155[a]	0.024	0.021	13.148	0.024	7.502	1	305	0.007

a. Predictors: (Constant). APOEcarE4

Node 2.

Descriptive Statistics

	Mean	Std. Deviation	N
Psychosis	16.58	12.051	163
MTHFR_677T-carriers	0.31	0.465	163
MTHFR_677C-carriers	0.16	0.367	163
MTHFR_1298A-carriers	0.10	0.307	163
MTHFR_1298C-carriers	0.48	0.501	163
COMT_V158M-A-cariers	0.26	0.439	163
COMT_V158M-G-cariers	0.21	0.412	163

Variables Entered/Removed[a]

Model	Variables Entered	Variables Removed	Method
1	MTHFR_677T-carriers	.	Stepwise (Criteria: Probability-of-F-to-enter <= 0.050. Probability-of-F-to-remove >= 0.100).

a. Dependent Variable: Psychosis

Model Summary

Model	R	R Square	Adjusted R Square	Std. Error of the Estimate	Change Statistics				
					R Square Change	F Change	df1	df2	Sig. F Change
1	0.201[a]	0.040	0.034	11.842	0.040	6.761	1	161	0.010

a. Predictors: (Constant). MTHFR_677T-carriers

Node 3.

Descriptive Statistics

	Mean	Std. Deviation	N
Psychosis	20.16	13.435	51
MTHFR_1298A-carriers	0.33	0.476	51
MTHFR_1298C-carriers	0.14	0.348	51
COMT_V158M-A-cariers	0.20	0.401	51
COMT_V158M-G-cariers	0.18	0.385	51

Variables Entered/Removed[a]

Model	Variables Entered	Variables Removed	Method
1	MTHFR_1298A-carriers	.	Stepwise (Criteria: Probability-of-F-to-enter <= 0.050. Probability-of-F-to-remove >= 0.100).

a. Dependent Variable: Psychosis

Model Summary

Model	R	R Square	Adjusted R Square	Std. Error of the Estimate	Change Statistics				
					R Square Change	F Change	df1	df2	Sig. F Change
1	0.315[a]	0.099	0.081	12.881	0.099	5.389	1	49	0.024

a. Predictors: (Constant). MTHFR_1298A-carriers

Table S5. Stepwise multiple logistic regression for "Hyperactivity" endophenotype

Node 1.

Descriptive Statistics

	Mean	Std. Deviation	N
Hyperactivity	11.41	9.155	308
APOE_E4-carriers	0.53	0.500	308
MTHFR_677T-carriers	0.31	0.461	308
MTHFR_677C-carriers	0.17	0.378	308
MTHFR_1298A-carriers	0.09	0.288	308
MTHFR_1298C-carriers	0.47	0.500	308
COMT_V158M-A-cariers	0.28	0.448	308
COMT_V158M-G-cariers	0.20	0.402	308

Variables Entered/Removed[a]

Model	Variables Entered	Variables Removed	Method
1	APOE_E4-carriers	.	Stepwise (Criteria: Probability-of-F-to-enter <= .050. Probability-of-F-to-remove >= .100).

a. Dependent Variable: Hyperactivity

Model Summary

Model	R	R Square	Adjusted R Square	Std. Error of the Estimate	Change Statistics				
					R Square Change	F Change	df1	df2	Sig. F Change
1	0.188[a]	0.035	0.032	9.007	0.035	11.214	1	306	0.001

a. Predictors: (Constant). APOE_E4-carriers

Node 2.

Descriptive Statistics

	Mean	Std. Deviation	N
Hyperactivity	9.79	8.528	163
MTHFR_677T-carriers	0.31	0.465	163
MTHFR_677C-carriers	0.16	0.367	163
MTHFR_1298A-carriers	0.10	0.307	163
MTHFR_1298C-carriers	0.48	0.501	163
COMT_V158M-A-cariers	0.26	0.439	163
COMT_V158M-G-cariers	0.21	0.412	163

Variables Entered/Removed[a]

Model	Variables Entered	Variables Removed	Method
1	MTHFR_677T-carriers	.	Stepwise (Criteria: Probability-of-F-to-enter <= .050. Probability-of-F-to-remove >= .100).

a. Dependent Variable: Hyperactivity

Model Summary

Model	R	R Square	Adjusted R Square	Std. Error of the Estimate	Change Statistics				
					R Square Change	F Change	df1	df2	Sig. F Change
1	0.180[a]	0.032	0.026	8.414	0.032	5.389	1	161	0.022

a. Predictors: (Constant). MTHFR_677T-carriers

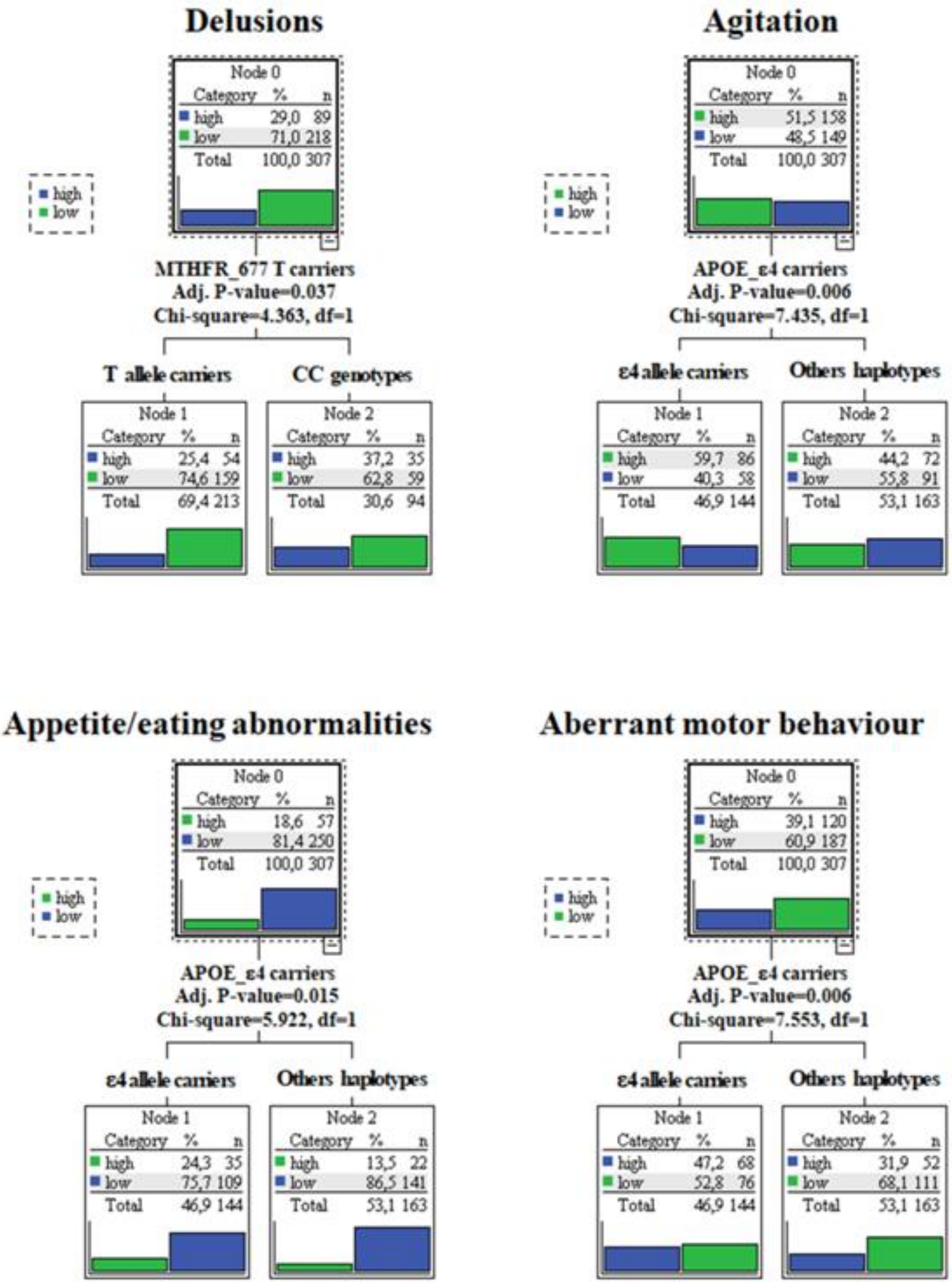

Figure S1. Exaustive CHAID data mining algorithm analysis results ("gene x gene" interaction) for single BPSD symptoms according to different values of "severity*frequencies"
